# A database of the coseismic effects following the 30 October 2016 Norcia earthquake in Central Italy

**DOI:** 10.1038/sdata.2018.49

**Published:** 2018-03-27

**Authors:** Fabio Villani, Riccardo Civico, Stefano Pucci, Luca Pizzimenti, Rosa Nappi, Paolo Marco De Martini, the Open EMERGEO Working Group, Fabio Villani, Riccardo Civico, Stefano Pucci, Luca Pizzimenti, Rosa Nappi, Paolo Marco De Martini, F. Agosta, G. Alessio, L. Alfonsi, M. Amanti, S. Amoroso, D. Aringoli, E. Auciello, R. Azzaro, S. Baize, S. Bello, L. Benedetti, A. Bertagnini, G. Binda, M. Bisson, A.M. Blumetti, L. Bonadeo, P. Boncio, P. Bornemann, S. Branca, T. Braun, F. Brozzetti, C.A. Brunori, P. Burrato, M. Caciagli, C. Campobasso, M. Carafa, F.R. Cinti, D. Cirillo, V. Comerci, L. Cucci, R. De Ritis, G. Deiana, P. Del Carlo, L. Del Rio, A. Delorme, P. Di Manna, D. Di Naccio, L. Falconi, E. Falcucci, P. Farabollini, J.P. Faure Walker, F. Ferrarini, M.F. Ferrario, M. Ferry, N. Feuillet, J. Fleury, U. Fracassi, C. Frigerio, F. Galluzzo, R. Gambillara, G. Gaudiosi, H. Goodall, S. Gori, L.C. Gregory, L. Guerrieri, S. Hailemikael, J. Hollingsworth, F. Iezzi, C. Invernizzi, D. Jablonská, E. Jacques, H. Jomard, V. Kastelic, Y. Klinger, G. Lavecchia, F. Leclerc, F. Liberi, A. Lisi, F. Livio, L. Lo Sardo, J.P. Malet, M.T. Mariucci, M. Materazzi, L. Maubant, F. Mazzarini, K.J.W. McCaffrey, A.M. Michetti, Z.K. Mildon, P. Montone, M. Moro, R. Nave, M. Odin, B. Pace, S. Paggi, N. Pagliuca, G. Pambianchi, D. Pantosti, A. Patera, E. Pérouse, G. Pezzo, L. Piccardi, P.P. Pierantoni, M. Pignone, S. Pinzi, E. Pistolesi, J. Point, L. Pousse, A. Pozzi, M. Proposito, C. Puglisi, I. Puliti, T. Ricci, L. Ripamonti, M. Rizza, G.P. Roberts, M. Roncoroni, V. Sapia, M. Saroli, A. Sciarra, O. Scotti, G. Skupinski, A. Smedile, A. Soquet, G. Tarabusi, S. Tarquini, S. Terrana, J. Tesson, E. Tondi, A. Valentini, R. Vallone, J. Van der Woerd, P. Vannoli, A. Venuti, E. Vittori, T. Volatili, L.N.J. Wedmore, M. Wilkinson, M. Zambrano

**Affiliations:** 1 Istituto Nazionale di Geofisica e Vulcanologia, Italy; 2 Università della Basilicata, Potenza 85100, Italy.; 3 Istituto Superiore per la Prevenzione e la Ricerca Ambientale (ISPRA), Roma 00144, Italy.; 4 Università degli Studi di Camerino, Camerino 62032, Italy.; 5 Università degli Studi “Gabriele D’Annunzio” di Chieti-Pescara, Centro Interuniversitario per l'Analisi Sismotettonica, Chieti 66100, Italy.; 6 Institut de Radioprotection et Sûreté Nucléaire, BERSSIN, 92262 Fontenay-aux-Roses, France.; 7 Aix-Marseille Université, CEREGE CNRS-IRD UMR 34, 13545 Aix en Provence, France.; 8 Università degli Studi dell’Insubria, Como 22100, Italy.; 9 Université de Strasbourg, CNRS, Lab Image Ville Environnement UMR 7362, Strasbourg, France.; 10 Università degli Studi di Roma "La Sapienza", Roma 00185, Italy.; 11 Institut de Physique du Globe de Paris, Sorbonne Paris Cité, Paris 75005, France.; 12 Agenzia nazionale per le nuove tecnologie, l’energia e lo sviluppo economico sostenibile, (ENEA), Roma 00196, Italy.; 13 Institute for Risk and Disaster Reduction, University College London, London WC1E 6BT, UK.; 14 Géosciences Montpellier, Université de Montpellier CNRS-UMR 5243, France.; 15 University of Leeds, Leeds LS2 9JS, UK.; 16 Université Grenoble Alpes, Université Savoie Mont Blanc, CNRS, IRD, IFSTTAR, ISTerre, Saint-Martin-d'Hères 38400, Grenoble, France.; 17 Birkbeck University of London, London WC1E 7HX, UK.; 18 Université Côte d’Azur, CNRS, Observatoire de la Côte d’Azur, IRD, Géoazur, Valbonne 06560, France.; 19 Università degli Studi di Cassino e del Lazio Meridionale, DICeM, Cassino 03043, Italy.; 20 Université de Strasbourg, CNRS, Institut de Physique du Globe de Strasbourg UMR 7516, Strasbourg, France.; 21 Durham University, Durham DH1, UK.; 22 Università degli Studi di Chieti-Pescara, DiSPUTer Chieti, Chieti 66100, Italy.; 23 Consiglio Nazionale delle Ricerche, Istituto di Geoscienze e Georisorse (IGG), 50121 Firenze, Italy.; 24 Università degli Studi di Perugia, Perugia 06123, Italy.; 25 SOGIN, Roma 00185, Italy.; 26 Geospatial Research Ltd, Durham DH1 4EL, UK.

**Keywords:** Tectonics, Seismology

## Abstract

We provide a database of the coseismic geological surface effects following the Mw 6.5 Norcia earthquake that hit central Italy on 30 October 2016. This was one of the strongest seismic events to occur in Europe in the past thirty years, causing complex surface ruptures over an area of >400 km^2^. The database originated from the collaboration of several European teams (Open EMERGEO Working Group; about 130 researchers) coordinated by the Istituto Nazionale di Geofisica e Vulcanologia. The observations were collected by performing detailed field surveys in the epicentral region in order to describe the geometry and kinematics of surface faulting, and subsequently of landslides and other secondary coseismic effects. The resulting database consists of homogeneous georeferenced records identifying 7323 observation points, each of which contains 18 numeric and string fields of relevant information. This database will impact future earthquake studies focused on modelling of the seismic processes in active extensional settings, updating probabilistic estimates of slip distribution, and assessing the hazard of surface faulting.

## Background & Summary

The central Apennines are characterized by high seismogenic potential, mostly due to shallow (5–15 km) normal-faulting earthquakes with magnitudes up to M 6.5-7 (ref. 1). The main driving mechanisms invoked for seismogenesis are the extensional regime and the large-scale uplift affecting the region since the Quaternary^2^. Analysis of geodetic data suggests that the axis of the central Apennines is undergoing NE-SW striking extension at a rate of ∼1–2.5 mm/yr^3^, with relative vertical peak velocities of 2.5–3.0 mm/yr over ~100 km wavelengths^4^. Paleoseismic evidence^5^ of surface faulting highlights the primary role in seismic activity played by a compound network of normal faults^6–8^. However, due to the tectonic complexity of the Apennines and the centennial/millennial recurrence times of the largest seismic events, only a few cases of clear surface faulting were documented systematically in Italy. The most relevant previously documented surface ruptures were the 1915 Mw 7.1 Avezzano^9–12^ and the 1980 Mw 6.9 Campania-Basilicata earthquakes^13,14^, both of which had ruptures that were several kilometres long and surface offsets of up to ∼1 m. In these events, modern advances in survey techniques including digital shared recording methods and photogrammetry (Structure from Motion) were not available. In both cases, the seismogenic significance of the extensive rupture length and surface offset in these earthquakes was only appreciated later through geologic, geomorphic, and paleoseismic studies. Before the events of 2016–2017, the central Apennines were struck by several normal-faulting earthquakes in the past thirty years. Two events ruptured the ground with offsets of a few centimetres: the Mw 6.0 (ref. 15) 1997 Colfiorito^16–18^ and the Mw 6.1 (ref. 19) 2009 L’Aquila earthquakes^20,21^.

**Figure 1:**
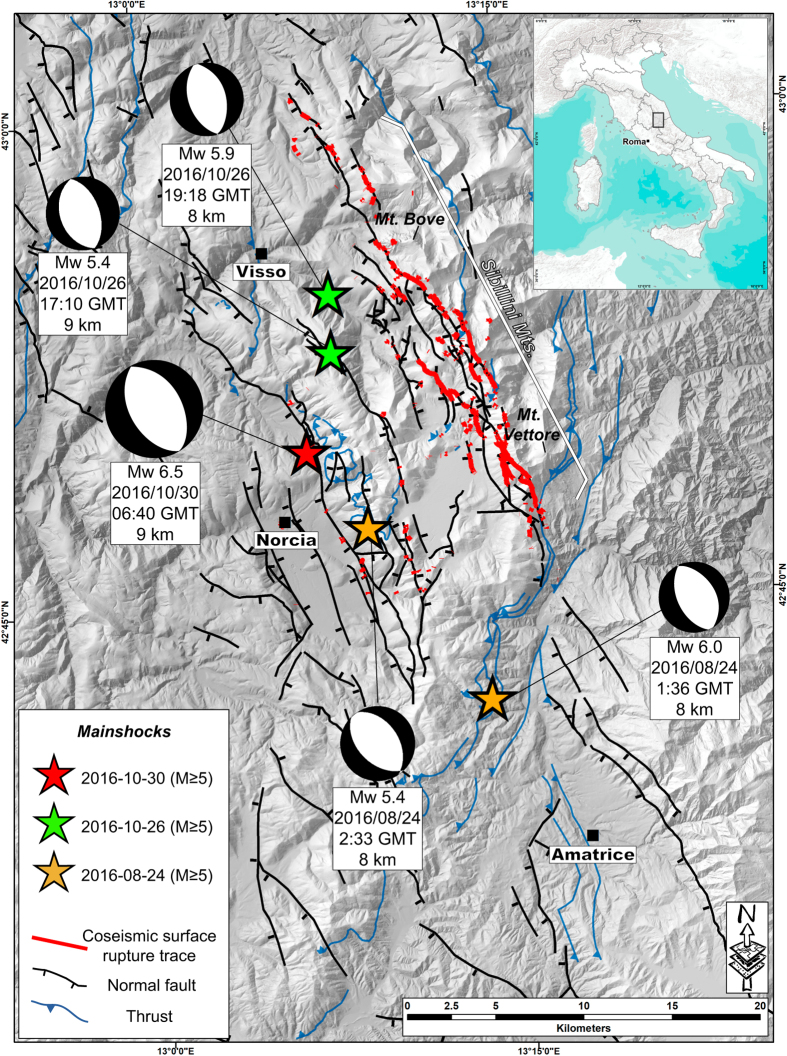
Geology of the 30 October 2016 M_w_ 6.5 Norcia earthquake area. Simplified structural scheme of the central Apennines inner zone, showing the main Quaternary normal faults (black lines, tick on downthrown side), Miocene-Pliocene thrusts (blue lines), the location of the M>5 events of the 2016-2017 seismic sequence, together with their focal mechanisms (available at http://cnt.rm.ingv.it/tdmt). The thin red lines are the mapped coseismic surface ruptures following the 30 October 2015 M_w_ 6.5 Norcia earthquake along which the data on coseismic ruptures discussed in this paper have been collected.

The 30 October 2016 Mw 6.5 earthquake (hereinafter Norcia earthquake) is the largest event in the seismic sequence^22,23^ that began on the 24 August 2016 (hereinafter Amatrice earthquake), with an Mw 6.0 earthquake causing 299 fatalities and heavy damage in Amatrice and surrounding villages (Figure 1). Thousands of aftershocks including several M≥5 events (e.g. Mw 5.9 on 26 October, close to Visso) occurred along a >50 km-long fault system. Following the Amatrice earthquake, surface faulting was documented in detail along the ~5.2 km rupture length with an average of ~13 cm of displacement^24–26^. However only sparse surface data were collected from the 26 October event due to the very limited time before the following quake, and these data are inconclusive for the total rupture length and average displacement. The Norcia earthquake resulted in an impressive system of surface ruptures that overprinted those of the previous two main 2016 surface-faulting events mentioned above.

The characterization of coseismic surface effects is crucial in earthquake geology, as it may unravel surface rupture propagated from seismogenic depth during the earthquake, its geometry and displacement amount. Such observations support studies on the mechanics of earthquake faulting, and are used to refine modelling of the seismic sources based on joint inversion of geophysical datasets (including strong motion recordings, GPS time-series and InSAR images).

The EMERGEO Working Group of the Istituto Nazionale di Geofisica e Vulcanologia (INGV) coordinated the field surveys performed with the contribution of about 130 European earth scientists. This joint venture gave rise to the Open EMERGEO Working Group, the largest scientific collaborative effort following a big earthquake in Europe. This cooperation was necessary for several reasons: 1) the Norcia earthquake coseismic effects covered an area >400 km^2^ wide, with local elevations exceeding >2200 m a.s.l. (Sibillini Mts.); 2) the forthcoming winter required an urgent survey of freshly-exposed ruptures; 3) destruction throughout the epicentral region was so intense that any survey required a coordination by INGV under the agreement with the Italian Civil Protection. In this respect, we highlight how science, best products, best practices, and operations benefit enormously from the cooperation among experts, in particular within the European context.

Surveys started within hours of the mainshock, aimed at documenting surface faulting and secondary coseismic phenomena^27^. This led to the recognition and mapping of the total extent of the surface ruptures, their geometry, kinematics and associated displacement. Here we summarize the most significant results in a concise and objective database, suitable for further studies.

Surface-rupturing earthquakes provide new data for characterising earthquake scaling laws, which are fundamental for seismic hazard analyses. Compilations of historical and paleoseismic events relating magnitude with rupture length and displacement^28–30^ can be increasingly improved with present-day observations. Our extensive and accurate database is essential in the perspective of better assessment of Fault Displacement Hazard and the building of an updated and worldwide database supporting this (INQUA project 2016–2019 SURFACE—SURface FAulting Catalogue Earthquakes; http://www.earthquakegeology.com/index.php?page=projects&s=4).

## Methods

**Figure 2:**
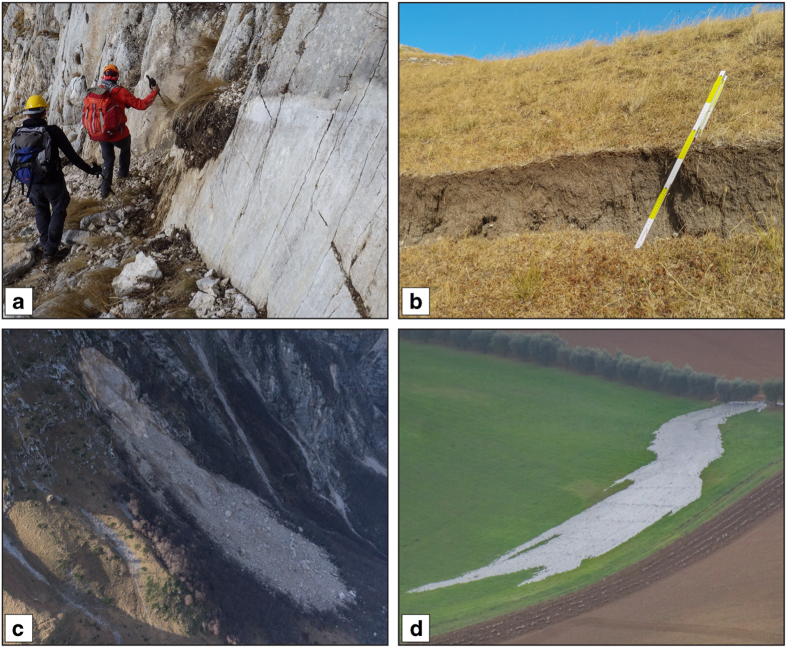
Examples of coseismic surface geological effects. (**a**) “coseismic ribbon on bedrock”: detail of surface rupture along the Mt. Vettore fault with local throw exceeding 1.80 m (persons for scale; location 13.2545° E, 42.8172° N); (**b**) “coseismic rupture with offset”, here affecting unconsolidated soils (ruler for scale; approximate location: 13.2211° E, 42.8958° N); (**c**) “coseismic sliding”: huge coseismic rockfall along the western slope of Mt. Bove (trees for scale; helicopter view, approximate location 13.2586° E, 42.8467° N); “coseismic mud volcanism” (trees for scale; helicopter view, approximate location 13.521° E, 43.040° N).

The survey of geological coseismic effects on the ground-surface following the Norcia earthquake was carried out according to classical morphotectonic and structural geology methods. Our approach focused on systematic surveys of the epicentral region searching for and documenting in detail any geomorphic feature related to coseismic surface displacement, such as: newly-formed ruptures affecting soils and loose deposits; fresh fault scarps; other indicators of tectonic disturbance such as hydrogeological anomalies directly or indirectly related to fault offset (spring discharge changes, stream deflections), or shaking (large landslides and mud volcanism). Figure 2 reports some representative pictures of the most important observed coseismic effects.

The main challenges were to investigate a >400 km^2^-wide area in a short time during an ongoing seismic sequence, and to avoid collecting data where there was modification by surface processes. We took advantage of available detailed fault and geologic mapping of the Sibillini Mts. reported in previous works^16,31–35^ including the 1:10,000 scale geological maps provided by Regione Umbria (available at: http://geo.umbriaterritorio.it/umbriageo/atlante/) and Regione Marche (available at: http://www.ambiente.marche.it/Territorio/Cartografiaeinformazioniterritoriali). The Mt. Vettore – Mt. Bove Fault-System (VBFS), clearly mapped in previous work as a ∼30 km-long Quaternary normal fault-system, shows evidence of recent surface faulting events^36^, and the VBFS proved to be the causative fault of the Norcia earthquake^23^: indeed, the large majority of coseismic surface ruptures was found along the previously mapped fault strands of the VBFS.

A large-scale detection of the surface rupture extent (about 25 km) and of its geometric arrangement were first established by means of a preliminary helicopter flight (31 October 2016). This was crucial for planning the early field surveys and for addressing the subsequent geological research on the main ruptures. Priorities were set to quickly recognize and document geomorphic features that: 1) were likely to undergo change due to surface processes reworking (e.g. possible weathering due to snowfall/rain), and 2) posed a potential threat to people (e.g. coseismic ruptures in close proximity or intersecting roads/infrastructures, or incipient large landsliding and rock-fall along steep slopes). Three helicopter surveys were carried out twenty days after the earthquake. Though delayed by poor weather conditions at higher elevations, these surveys provided more than 11,000 oblique-view photos spanning the entire rupture surface. These pictures revealed the occurrence of additional strands of surface ruptures that had not been recognized in the early days, and that were carefully investigated at a later date. About 80% of the records reported in our database was collected within one month from the occurrence of the 30 October mainshock, and nearly 90% of the database was completed by the end of December 2016: the remaining surveys were done between May and July 2017. This implies that our measurements of coseismic surface offset may potentially include some afterslip.

To perform our field surveys we adopted both analogue and digital instruments. We used precision analogue geological compasses, clinometers and digital mobile devices to measure the orientations of planes (dip angle, dip direction, strike) and lines (rake, slip vector trend, slip vector plunge) of interest. We used measuring tapes, rulers and rods for linear measurements (e.g. length of surface trace, opening, vertical offset and net offset of the ruptures). We collected strike measurements of coseismic ruptures with appreciable vertical offset adopting the right hand rule (i.e. looking to the strike direction, the plane dips to the right).

Most of the measurements were performed using digital mobile devices equipped with specific software (Rocklogger© mobile app, www.rockgecko.com) employing accelerometer, gyroscope, electronic magnetometer, and GPS to determine the exact orientation and position in space. This allowed for quick and accurate structural data collection, and real-time sharing with the EMERGEO Working Group main office at INGV in Rome^37^. Such real-time data sharing, together with GPS track logging, proved to be of utmost importance in the daily planning of surveys.

### Database preparation

The main goal of this work was to organize in a common and easy-to-use database field observations containing quantitative measurements and notes collected by different geologists, sometimes with quite different approaches and written in different languages (i.e. Italian, English and French). Therefore, the first stage of the workflow consisted of careful screening of the collected data, which were reported to the EMERGEO Working Group coordinators in the form of spreadsheet and comma-separated values (CSV) files, according to the formatting protocol established at the beginning of the field surveys by the EMERGEO Working Group guidelines^37^.

Subsequently, the individual reports in the form of CSV files were input to a Visual Basic macro (MACRO_EMERGEO.xlsm, provided in the Supplementary Material) in order to merge them in a unique spreadsheet. After the first stage of individual reports analysis, we retained a minimum number of fields that were able to synoptically describe all of the observation points, and removed the unnecessary and/or subjective comments. Since some data were not associated with an absolute elevation, we extracted elevation values from a 10-m grid digital elevation model (DEM)^38^ for the whole dataset.

Firstly, the macro parses the CSV- format files matching the standard protocol and converts them into a Microsoft Excel spreadsheets file (XLS). Subsequently, it merges each single XLS file creating an unique table, assigning a progressive ID to each record based on the acquisition data/time (yyyy-mm-dd hh:mm:ss format), and contemporaneously exports a standard Keyhole Markup Language (KMZ) file. These KMZ files were also used during the seismic crisis for daily planning of field surveys, and to check the updated coverage of the mapped sections with respect to the overall extent of surface ruptures recognized by helicopter flights.

The output spreadsheet needed some further editing by removing scattered data with erroneous coordinates falling outside the surveyed area and marked with low quality indexes provided by the digital mobile devices. Therefore, it was converted in an ESRI shapefile (.shp) on ArcGIS© platforms that can be plotted, explored and edited by standard SQL queries. During this conversion procedure, some empty fields (i.e. no data or NULL value) may erroneously be converted into zeroes. Therefore, since the ESRI shapefile does not support NULL values, we made the necessary editing and conversion using the software QGIS 2.18© (http://qgis.org/it/site/about/index.html). After final editing, the .shp file was finally converted into the text (TXT) file provided in this work.

## Data Records

The complete dataset in the present study was uploaded in the *Pangaea* repository (Data Citation 1): it is a TXT file (Villani-*etal*_2017.tab) consisting of 7323 records organized into 18 fields. Each record describes a single observation point. The fields have a name and a short name, and they are described as follows:

1. **ORDINAL NUMBER** (short name: Ord No): integer type variable defining the object identifier;
2. **DATE/TIME** (short name: date/Time): date type variable indicating the date of data collection, in the format yyyy-mm-dd;
3. **LATITUDE** (short name: Latitude six decimal places): double type variable indicating the latitude of the observation, in decimal degrees (dd.mmmmmm) within a WGS_1984 Geographic Coordinate System;
4. **LONGITUDE** (short name: Longitude; six decimal places): double type variable indicating the longitude of the observation point, in decimal degrees (dd.mmmmmm) within a WGS_1984 Geographic Coordinate System;
5. **ELEVATION** (short name: Elevation; no decimal place): double type variable indicating the absolute elevation of the observation point in meters above the sea level; the elevation is extracted from a 10- m grid DEM described in the Methods Section;
6. **Observation** (short name: Obs): text variable indicating the basic category of the geological surface effect observed; for simplicity, only six main categories are defined, as follows: “Coseismic rupture with offset” (ground break displaying a perceivable vertical offset of the ground surface >1 cm); “Coseismic fracture” (ground break with no perceivable vertical offset, i.e. << 1 cm); “Coseismic ribbon on bedrock” (fresh stripe running at the base of a bedrock fault scarp due to coseismic exhumation); “Coseismic slip vector” (lineation defining the coseismic net displacement occurred on a fault plane or along a ground break); “Coseismic sliding” (generic landslide of ascertained coseismic origin); “Coseismic mud volcanism” (mud volcanism phenomena related to the earthquake); “Coseismic dislocation” (warping of the ground surface with no perceivable fracturing);
7. **Rock type** (short name: Rock): text type variable including the synthetic description of the lithological nature of the substratum where the coseismic effect was reported; the original note of the field operator has been slightly modified, simplified and/or translated from Italian or French to English;
8. **Angle** (short name: Angle; no decimal places): double type variable indicating the angle of dip of a rupture or sliding surface, measured in degrees;
9. **Direction** (short name: Direction; no decimal places): double type variable indicating the direction of dip of a rupture or sliding surface with respect to the North, measured in degrees;
10. **Strike** (short name: Strike; no decimal places): double type variable indicating the azimuth angle of a rupture or sliding surface with respect to the North, measured in degrees;
11. **Length** (short name: l; one decimal place): double type variable indicating the length measured in meters of a rupture or sliding surface;
12. **Opening** (short name: Opening; no decimal places): double type variable indicating the aperture of a rupture or sliding surface measured in centimetres, orthogonal to the fracture walls;
13. **Throw** (short name: Throw; one decimal place): double type variable indicating the vertical separation of a coseismic rupture measured in centimetres;
14. **Offset** (short name: Offset; one decimal place): double type variable indicating the net displacement of a coseismic rupture evaluated directly on a bedrock fault plane or using piercing points when the rupture affects unconsolidated sediments measured in centimetres;
15. **Rake** (short name: Rake; no decimal places): double type variable indicating the angle of the slip lineation on the fault plane measured in degrees (in the 0°-180° range): note that some geologists used the Aki and Richards^39^ annotation, where normal motion is indicated with negative rake values;
16. **Vector** (short name: Vec; no decimal places): double type variable indicating the direction of the slip lineation in degrees, measured clockwise with respect to the north (range 0°-360°);
17. **Vector** (short name: Vec; no decimal places): double type variable indicating the plunge of the slip lineation in degrees, measured with respect to the horizontal (range 0°—90°);
18. **Group** (short name: Group): text type variable indicating the geologists’ team who collected the data.

**Figure 3:**
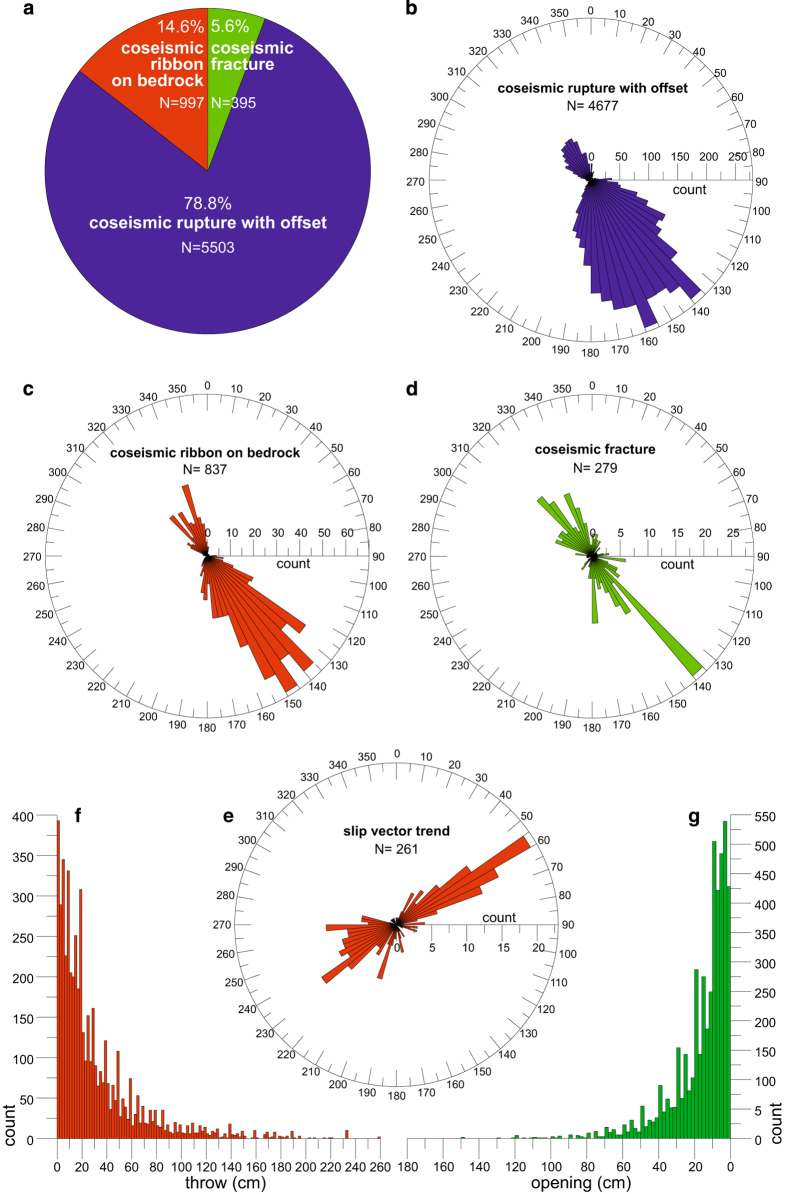
Statistical properties of the coseismic surface ruptures. (**a**) pie diagram indicating the relative proportion of the main types of measured coseismic ruptures; (**b**) rose diagram of the coseismic ruptures with offset strike (bin size 5°); (**c**) rose diagram of the coseismic ribbons on bedrock strike (bin size 5°); (**d**) rose diagram of the coseismic fractures strike (bin size 5°); (**e**) rose diagram of the coseismic slip vectors trend; **f**) frequency plot histogram of the coseismic surface throw (bin size 2 cm); (**g**) frequency plot histogram of the coseismic surface opening (x-axis flipped, bin size 2 cm; note that the horizontal and vertical axes in panels f and g are scaled differently).

Table 1 reports as an example six records from the database. Figure 3 shows the main geometric properties of the coseismic surface data, in order to provide a measure of the data partitioning and the occurrence of significant modal peaks in their frequency distribution. The pie diagram in Figure 3a displays a total of 6895 measurements of surface ruptures, subdivided in the following categories: 395 “coseismic fractures” (about 5.6%), 5503 “coseismic ruptures with offset” (about 79.8%) and 997 “coseismic ribbon on bedrock” (about 14.6%). A large sub-set of these measurements has azimuthal information. In particular, 4677 measurements of coseismic ruptures with offset indicate the occurrence of two directional peaks of strike at N135°-140° and N155°-160°, respectively (Figure 3b). Similarly, the strike of coseismic ribbons on bedrock (837 measurements) displays two modal peaks at N135°-140° and N145°-150°, respectively (Figure 3c), whereas the strike of coseismic fractures (279 measurements) displays one sharp peak at N135°-140° (Figure 3d). Thus, the large majority of the surface ruptures parallels the mapped strands of the VBFS, and the strike of the nodal planes of the focal mechanisms shown in Figure 1. Consistently with this geometric arrangement, the trend of the measured slip vectors (261 measurements; Figure 3e) displays two peaks at N55°-60° and at N230°-235°, which are almost at right angles to the surface ruptures. This indicates the occurrence of both SW-dipping and NE-dipping ruptures characterized by a dominant dip-slip motion.

The two frequency histogram plots in Figures 3f and g indicate that measured surface throws and opening are characterized by complex multi-modal and strongly skewed distributions. The “coseismic ruptures with offset” have mean and maximum throw of 24 cm and 221 cm, respectively. The “coseismic ribbon on bedrock” measurements have an average and maximum throw of 69 cm and 260 cm, respectively. Overall, the surface ruptures affecting loose deposits and/or running at the base of bedrock fault planes have average and maximum throws of 31 cm and 260 cm, respectively. The frequency distribution of rupture opening has an average value of 17 cm and a maximum value of 182 cm.

## Technical Validation

Both qualitative and quantitative factors contribute to the uncertainty affecting the collected field measurements reported in the database. Qualitative uncertainty factors include, but are not limited to, environmental conditions, representativeness of the measured object and instrument maintenance and operation. Some factors affecting quantitative uncertainty are environmental conditions, instrument precision and accuracy, instrument calibration, equipment maintenance and field operations.

We reduced the impact of those qualitative and quantitative uncertainty factors through the development of standard operating procedures. For instance, significant errors may be introduced in the collection of the observation points if the target to be measured is poorly defined and/or affected by personal interpretations. To compensate for these errors, we reached a consensus on the features of interest to be measured, on the conventions for how to specify the orientations of lines and planes and on how to perform linear measurements.

With regard to the measurement of planes and lines orientation, in order to diminish the systematic errors produced by uncalibrated or poorly maintained instruments 1) we checked that the analogue compasses were correctly adjusted for the magnetic declination (which, at the latitude of the study area, is about 3°), and 2) the EMERGEO Working Group personnel used the same digital mobile devices (Samsung Galaxy Note 2), which were repeatedly calibrated following the standard procedure suggested by the Rocklogger© mobile app before each survey.

One of the great advantages of using digital mobile devices for field surveys is that one can take several measurements of the same feature in a time span of a few seconds, providing readings that will be statistically more representative of the geologic object than a single measurement taken with an analogue compass. By comparing the results of the orientation measurements of lines and planes collected at the same location by different operators, we found that the average dispersion in the measured values was±5°, which is an adequate accuracy for a meaningful measurement of the geological features. The measurement of fracture opening and vertical offset of the surface ruptures has a precision of about 1 cm, which is common for the standard tapes we used and the rough micro-topography of the ground surface. Therefore, ground ruptures with very small offset (a few cm) are proportionately affected by errors in the order of 10–30%, whereas ruptures with large offsets (> 1 m) bear error of less than 5%.

GPS-enabled digital mobile devices have position accuracy with a median horizontal error of position fixes between 5.0 m and 8.5 m^40^, a value comparable to that observed for our measurements points. We used high-resolution images obtained from ESRI World Imagery (http://services.arcgisonline.com/ArcGIS/rest/services/World_Imagery/MapServer) together with very recent post-seismic Google Maps imagery as a basemap to visually cross-check the observation points in ESRI ArcGIS, and thus remove a few misplaced data points. We also performed a comprehensive check to delete duplicate records and other typing errors.

During the seismic crisis, the real-time sharing of the collected data enabled us to quickly perform a preliminary quality control with a nearly daily frequency: in the case we pointed out the occurrence of some controversial or doubt measurements (such as outliers in the surface slip distribution, unexpected dip direction of ruptures, or sites exhibiting features of problematic interpretation), we promptly organized specific surveys to check again for the consistency of the measurements and the nature of the coseismic effects. This constant feedback between field geologists and the central coordination exerted by the EMERGEO Working Group in Rome is one of the main points characterizing the overall validation of such a large set of data following a strong surface-rupturing earthquake.

## Additional information

**How to cite this article**: Villani F. *et al.* A database of the coseismic effects following the 30 October 2016 Norcia earthquake in Central Italy. *Sci. Data* 5:180049 doi: 10.1038/sdata.2018.49 (2018).

**Publisher’s note**: Springer Nature remains neutral with regard to jurisdictional claims in published maps and institutional affiliations.

## Supplementary Material



Supplementary Information

## Figures and Tables

**Table 1:** An example of 6 records extracted from the database.

**Ord No**	**Date/Time**	**Latitude**	**Longitude**	**Elevation [m a.s.l.]**	**Obs**	**Rock**	**Angle [deg]**	**Direction [deg]**	**Strike [deg]**	**l [m]**	**Opening [cm]**	**Throw [cm]**	**Offset [m]**	**Rake [deg]**	**Vec [deg] (slip vector, trend)**	**Vec [deg] (slip vector, plunge)**	**Group**
1485	2016-11-10	42.859138	13.206234	1453	Coseismic slip vector	Limestone/Debris	42	51	321				0.75	−85	44	42	Emergeo_WG
1486	2016-11-10	42.859258	13.205873	1430	Coseismic slip vector	Limestone/Debris	37	55	325				0.69	−95	61	37	Emergeo_WG
1485	2016-11-10	42.859138	13.206234	1453	Coseismic slip vector	Limestone/Debris	42	51	321				0.75	−85	44	42	Emergeo_WG
1486	2016-11-10	42.859258	13.205873	1430	Coseismic slip vector	Limestone/Debris	37	55	325				0.69	−95	61	37	Emergeo_WG
5770	2016-12-20	42.854591	13.101894	922	Coseismic rupture with offset	Soil/debris	90	45	315	12.6	5	1					ENEA
5771	2016-12-20	42.854605	13.10111	904	Coseismic rupture with offset	Soil/debris	85	248	158	1.5	16	16					ENEA
