# Publisher Correction: A database of the coseismic effects following the 30 October 2016 Norcia earthquake in Central Italy

**DOI:** 10.1038/s41597-018-0001-6

**Published:** 2019-02-19

**Authors:** Fabio Villani, Riccardo Civico, Stefano Pucci, Luca Pizzimenti, Rosa Nappi, Paolo Marco De Martini, the Open EMERGEO Working Group, Fabio Villani, Riccardo Civico, Stefano Pucci, Luca Pizzimenti, Rosa Nappi, Paolo Marco De Martini, F. Agosta, G. Alessio, L. Alfonsi, M. Amanti, S. Amoroso, D. Aringoli, E. Auciello, R. Azzaro, S. Baize, S. Bello, L. Benedetti, A. Bertagnini, G. Binda, M. Bisson, A. M. Blumetti, L. Bonadeo, P. Boncio, P. Bornemann, S. Branca, T. Braun, F. Brozzetti, C. A. Brunori, P. Burrato, M. Caciagli, C. Campobasso, M. Carafa, F. R. Cinti, D. Cirillo, V. Comerci, L. Cucci, R. De Ritis, G. Deiana, P. Del Carlo, L. Del Rio, A. Delorme, P. Di Manna, D. Di Naccio, L. Falconi, E. Falcucci, P. Farabollini, J. P. Faure Walker, F. Ferrarini, M. F. Ferrario, M. Ferry, N. Feuillet, J. Fleury, U. Fracassi, C. Frigerio, F. Galluzzo, R. Gambillara, G. Gaudiosi, H. Goodall, S. Gori, L. C. Gregory, L. Guerrieri, S. Hailemikael, J. Hollingsworth, F. Iezzi, C. Invernizzi, D. Jablonská, E. Jacques, H. Jomard, V. Kastelic, Y. Klinger, G. Lavecchia, F. Leclerc, F. Liberi, A. Lisi, F. Livio, L. Lo Sardo, J. P. Malet, M. T. Mariucci, M. Materazzi, L. Maubant, F. Mazzarini, K. J. W. McCaffrey, A. M. Michetti, Z. K. Mildon, P. Montone, M. Moro, R. Nave, M. Odin, B. Pace, S. Paggi, N. Pagliuca, G. Pambianchi, D. Pantosti, A. Patera, E. Pérouse, G. Pezzo, L. Piccardi, P. P. Pierantoni, M. Pignone, S. Pinzi, E. Pistolesi, J. Point, L. Pousse, A. Pozzi, M. Proposito, C. Puglisi, I. Puliti, T. Ricci, L. Ripamonti, M. Rizza, G. P. Roberts, M. Roncoroni, V. Sapia, M. Saroli, A. Sciarra, O. Scotti, G. Skupinski, A. Smedile, Anne Socquet, G. Tarabusi, S. Tarquini, S. Terrana, J. Tesson, E. Tondi, A. Valentini, R. Vallone, J. Van der Woerd, P. Vannoli, A. Venuti, E. Vittori, T. Volatili, L. N. J. Wedmore, M. Wilkinson, M. Zambrano

**Affiliations:** 1 Istituto Nazionale di Geofisica e Vulcanologia, Roma, Italy; 2 Università della Basilicata, Potenza, 85100 Italy; 3 Istituto Superiore per la Prevenzione e la Ricerca Ambientale (ISPRA), Roma, 00144 Italy; 4 Università degli Studi di Camerino, Camerino, 62032 Italy; 5 Università degli Studi “Gabriele D’Annunzio” di Chieti-Pescara, Centro Interuniversitario per l’Analisi Sismotettonica, Chieti, 66100 Italy; 6 Institut de Radioprotection et Sûreté Nucléaire, BERSSIN, 92262 Fontenay-aux-Roses, France; 7 Aix-Marseille Université, CEREGE CNRS-IRD UMR 34, 13545 Aix en Provence, France; 8 Università degli Studi dell’Insubria, Como, 22100 Italy; 9 Université de Strasbourg, CNRS, Lab Image Ville Environnement UMR, 7362 Strasbourg, France; 10 Università degli Studi di Roma “La Sapienza”, Roma, 00185 Italy; 11 Institut de Physique du Globe de Paris, Sorbonne Paris Cité, Paris, 75005 France; 12 Agenzia nazionale per le nuove tecnologie, l’energia e lo sviluppo economico sostenibile, (ENEA), Roma, 00196 Italy; 13 Institute for Risk and Disaster Reduction, University College London, London, WC1E 6BT UK; 14 Géosciences Montpellier, Université de Montpellier, Montpellier, CNRS-UMR 5243 France; 15 University of Leeds, Leeds, LS2 9JS UK; 16 Université Grenoble Alpes, Université Savoie Mont Blanc, CNRS, IRD, IFSTTAR, ISTerre, Saint-Martin-d’Hères, 38400 Grenoble, France; 17 Birkbeck University of London, London, WC1E 7HX UK; 18 Université Côte d’Azur, CNRS, Observatoire de la Côte d’Azur, IRD, Géoazur, Valbonne, 06560 France; 19 Università degli Studi di Cassino e del Lazio Meridionale, DICeM, Cassino, 03043 Italy; 20 Université de Strasbourg, CNRS, Institut de Physique du Globe de Strasbourg UMR, 7516 Strasbourg, France; 21 Durham University, Durham, DH1 UK; 22 Università degli Studi di Chieti-Pescara, DiSPUTer Chieti, Chieti, 66100 Italy; 23 Consiglio Nazionale delle Ricerche, Istituto di Geoscienze e Georisorse (IGG), 50121 Firenze, Italy; 24 Università degli Studi di Perugia, Perugia, 06123 Italy; 25 SOGIN, Roma, 00185 Italy; 26 Geospatial Research Ltd, Durham, DH1 4EL UK

Correction to: *Scientific Data* 10.1038/sdata.2018.49, published online 27 March 2018

In the original version of the Data Descriptor the surname of author Anne Socquet was misspelled. This has now been corrected in the HTML and PDF versions of the Data Descriptor.

Some authors were also not appropriately associated with their affiliations in the HTML version, due to formatting errors made by the publisher. This has now been corrected in the HTML version of the Data Descriptor, the affiliations in the PDF were correct from the time of publication.

