# Supplementary material for: A database of the coseismic effects following the 30 October 2016 Norcia earthquake in Central Italy
Source: Sci Data. 2018 Mar 27;5:180049. doi: 10.1038/sdata.2018.49 (PMC5870340; doi:10.1038/sdata.2018.49)
Supplement: Supplementary Information [file sdata201849-s2.docx]

**Supplementary material**

This supplementary information contains the Visual Basic code we used to parse the original raw field data and merge them into a unique spreadsheet. It is composed of three subroutines. We removed the original comments, which were written in Italian.

**Table 1.**

Visual basic code MACRO_EMERGEO.xlsm.

***Sub AUTO1_Normalizza_CSVXLS()***

*Dim Filename, Pathname, saveFileName, Myfile, textline, Var1, sCOL, sCELL, sLetter As String*

*Dim Position, iNu As Integer*

*Dim rgFound As Range*

*Dim wb As Workbook*

*Dim initialDisplayAlerts As Boolean*

*Pathname = ThisWorkbook.Path*

*Filename = Dir(Pathname & "\01_INPUT_CSVXLS\*.*")*

*Do While Filename <> ""*

*'************se sono CSV*

*If Right(Filename, 3) = "csv" Then*

*Set wb = Workbooks.Open(Filename:=Pathname & "\01_INPUT_CSVXLS\" & Filename, _*

*UpdateLinks:=False, Delimiter:=",")*

*saveFileName = Replace(Filename, ".csv", ".xls")*

*'************se sono XLS*

*ElseIf Right(Filename, 3) = "xls" Then*

*Set wb = Workbooks.Open(Filename:=Pathname & "\01_INPUT_CSVXLS\" & Filename, _*

*UpdateLinks:=False)*

*saveFileName = Filename*

*Else*

*GoTo NextIteration*

*End If*

*wb.CheckCompatibility = False*

*If Range("A1").Value <> "Datestamp" Then*

*Rows(1).EntireRow.Delete*

*Rows(1).EntireRow.Delete*

*Rows(1).EntireRow.Delete*

*End If*

*Myfile = Pathname & "\intestazioni_campi.ini" '*

*Open Myfile For Input As #1*

*Line Input #1, textline*

*iNu = 1*

*Position = InStr(iNu, textline, Chr(9))*

*Do While Position > 0*

*Var1 = Mid(textline, 1, Position - 1)*

*Set rgFound = Range("1:1").Find(Var1)*

*If rgFound Is Nothing Then*

*sLetter = ConvertToLetter(iNu)*

*sCOL = sLetter & ":" & sLetter*

*sCELL = sLetter + "1"*

*Columns(sCOL).Insert Shift:=xlToRight, CopyOrigin:=xlFormatFromLeftOrAbove*

*Range(sCELL).Value = Var1*

*End If*

*textline = Mid(textline, Position + 1, 1000)*

*Position = InStr(1, textline, Chr(9))*

*iNu = iNu + 1*

*Loop*

*wb.SaveAs Filename:=Pathname & "\02_OUTPUT_XLS_SINGOLI\" & saveFileName, _*

*FileFormat:=xlExcel8, Password:="", WriteResPassword:="", _*

*ReadOnlyRecommended:=False, CreateBackup:=False*

*wb.Close SaveChanges:=False*

*Close #1*

*NextIteration:*

*Filename = Dir()*

*Loop*

*Close #1*

*Application.DisplayAlerts = initialDisplayAlerts*

*End Sub*

***Sub AUTO2_CREA_XLS_GEN()***

*Application.DisplayAlerts = False*

*Dim Filename, Filename2, Pathname, saveFileName, Myfile, textline, Var1, sCOL, sCELL1, sCELL2, sCELL1a, sCELL2a, sCELL3, sLetter As String*

*Dim Position, iNu, Rdivy, Rdivx, Rdivz, Rdivw, riga As Integer*

*Dim rgFound As Range*

*Dim wb1, wb2 As Workbook*

*Pathname = ThisWorkbook.Path*

*Filename2 = Dir(Pathname & "\03_OUTPUT_XLS_GENERALE\*.xls")*

*Filename = Dir(Pathname & "\02_OUTPUT_XLS_SINGOLI\*.xls")*

*Set wb1 = Workbooks.Open(Filename:=Pathname & "\03_OUTPUT_XLS_GENERALE\" & Filename2, _*

*UpdateLinks:=False)*

*Myfile = Pathname & "\id_obs.ini"*

*Open Myfile For Input As #1*

*Line Input #1, textline*

*Close #1*

*Rdivz = Range("A65356").End(xlUp).Row + 1*

*Do While Filename <> ""*

*If Len(Filename) > 29 Then*

*sTeam = Left(Filename, Len(Filename) - 29)*

*Else*

*sTeam = "Team?"*

*End If*

*wb1.Activate*

*Rdivx = Range("A65356").End(xlUp).Row + 1*

*sCELL1 = "c" & Rdivx*

*sCELL1a = "l" & Rdivx*

*Set wb2 = Workbooks.Open(Filename:=Pathname & "\02_OUTPUT_XLS_SINGOLI\" & Filename, _*

*UpdateLinks:=False)*

*Rdivy = Range("A65356").End(xlUp).Row + 1*

*sCELL2 = "h" & (Rdivy - 1)*

*sCELL2a = "af" & (Rdivy - 1)*

*ActiveSheet.Range("a2", sCELL2).Copy*

*wb1.Activate*

*ActiveSheet.Range(sCELL1).PasteSpecial xlPasteAll*

*wb2.Activate*

*ActiveSheet.Range("i2", sCELL2a).Copy*

*wb1.Activate*

*ActiveSheet.Range(sCELL1a).PasteSpecial xlPasteAll*

*ActiveWorkbook.Save*

*wb2.Close SaveChanges:=False*

*For riga = Rdivx To Rdivx + Rdivy - 3*

*Cells(riga, "a").Value = textline & "-" & (riga - 1)*

*Cells(riga, "b").Value = DateValue(Left(Cells(riga, "c").Value, 10))*

*Var1 = Cells(riga, "s").Value*

*If InStr(1, Var1, "Overturned") Then*

*'Stop*

*If Cells(riga, "j").Value <> "" Then*

*'Stop*

*Cells(riga, "k").Value = (Cells(riga, "j").Value - 180)*

*If Cells(riga, "k").Value < 0 Then*

*Cells(riga, "k").Value = (Cells(riga, "j").Value + 180)*

*End If*

*End If*

*Else*

*Cells(riga, "k").Value = Cells(riga, "j").Value*

*End If*

*Cells(riga, "AJ").Value = sTeam*

*Next riga*

*Filename = Dir()*

*Loop*

*'Stop*

*Rdivw = Range("A65356").End(xlUp).Row + 1*

*sCELL3 = "A" & Rdivz*

*Range(sCELL3, "AZ65356").Sort Key1:=Range("B1"), Order1:=xlAscending, Header:=xlNo, Key2:=Range("AJ1"), Order1:=xlAscending, Header:=xlNo, Key1:=Range("C1"), Order1:=xlAscending, Header:=xlNo*

*For riga = Rdivz To Rdivw - 1*

*Cells(riga, "a").Value = textline & "-" & (riga - 1)*

*Next*

*wb1.Close SaveChanges:=True*

*End Sub*

***Sub AUTO3_CREA_KML_FIELD()***

*Dim Filename, Myfile, Pathname, sID_obs, sTeam, sDatestamp, sGPSacc, sLat, sLon, sAlt As String*

*Dim Rdivx As Integer*

*Dim wb1 As Workbook*

*Pathname = ThisWorkbook.Path*

*Filename = Dir(Pathname & "\03_OUTPUT_XLS_GENERALE\*.xls")*

*Set wb1 = Workbooks.Open(Filename:=Pathname & "\03_OUTPUT_XLS_GENERALE\" & Filename, _*

*UpdateLinks:=False)*

*Filename = Left(Filename, Len(Filename) - 4)*

*Myfile = Pathname & "\04_OUTPUT_KML_FIELD\" & Filename & ".kml"*

*Open Myfile For Output As #2*

*Print #2, "<?xml version=" + Chr(34) + "1.0" + Chr(34) + " encoding=" + Chr(34) + "iso-8859-1" + Chr(34) + "?>"*

*Print #2, "<kml xmlns=" + Chr(34) + "http://earth.google.com/kml/2.0" + Chr(34) + ">"*

*Print #2, "<Document>"*

*'*********************************************

*Rdivx = Range("A65356").End(xlUp).Row + 1*

*For riga = 2 To Rdivx*

*sID_obs = CStr(Cells(riga, "A").Value)*

*sTeam = CStr(Cells(riga, "AJ").Value)*

*sDatestamp = CStr(Cells(riga, "C").Value)*

*sLat = CStr(Cells(riga, "E").Value)*

*sLon = CStr(Cells(riga, "F").Value)*

*sAlt = CStr(Cells(riga, "G").Value)*

*sGPSacc = CStr(Cells(riga, "H").Value)*

*Print #2, " "*

*Print #2, "<Placemark>"*

*Print #2, "<description><![CDATA["*

*Print #2, "<b>ID_obs: </b>" + sID_obs + "<BR>"*

*Print #2, "<b>Datestamp: </b>" + sDatestamp + "<BR>"*

*Print #2, "<b>Team: </b>" + sTeam + "<BR>"*

*Print #2, "<b>Lat: </b>" + sLat + "<BR>"*

*Print #2, "<b>Lon: </b>" + sLon + "<BR>"*

*Print #2, "<b>Alt: </b>" + sAlt + "<BR>"*

*Print #2, "<b>GPSacc: </b>" + sGPSacc + "<BR>"*

*Print #2, "]]> </description>"*

*Print #2, "<Point> <coordinates>" + sLon + "," + sLat + "</coordinates> </Point>"*

*Print #2, "</Placemark>"*

*Next riga*

*Print #2, "</Document></kml>"*

*Close #2*

*End Sub*

*Function ConvertToLetter(iCol As Integer) As String*

*Dim iAlpha As Integer*

*Dim iRemainder As Integer*

*iAlpha = Int(iCol / 27)*

*iRemainder = iCol - (iAlpha * 26)*

*If iAlpha > 0 Then*

*ConvertToLetter = Chr(iAlpha + 64)*

*End If*

*If iRemainder > 0 Then*

*ConvertToLetter = ConvertToLetter & Chr(iRemainder + 64)*

*End If*

*End Function*
